# Simultaneous Analysis of Wnt and NF-κB Signaling Pathways in Doxorubicin Sensitive and Methotrexate Resistant PLC/ PRF/5 Cells

**DOI:** 10.22074/cellj.2016.3845

**Published:** 2016-01-17

**Authors:** Nasrin Shojaie, Seyed Mahmood Ghaffari

**Affiliations:** Biochemistry Group, Institute of Biochemistry and Biophysics, University of Tehran, Tehran, Iran

**Keywords:** Doxorubicin, MDR, Methotrexate, NF-κB, Wnt

## Abstract

**Objective:**

Multi-drug resistance (MDR) is a controversial issue in traditional chemo- therapy of aggressive cancers, including hepatocellular carcinoma. The major cause of MDR is suggested to be the aberrant activation of the main signaling pathways such as Wnt and nuclear factor kappa-light-chain-enhancer of activated B cells (NF- κB) which have key roles in the maintenance of cancer stem cells (CSCs). Therefore, the evaluation of their alterations could be essential in chemo-resistant cancers such as Hepatocellular carcinoma. The main purpose of this study was to investigate the alteration of the mentioned pathways in the chemotherapy resistant cancer cells by assessing their major molecular parameters.

**Materials and Methods:**

In this experimental study, methylthiazol tetrazolium (MTT) assay, acridine orange/ethidium bromide (AO/EtBr) and Hoechst 33342 staining, DNA fragmentation and colony formation methods were employed to investigate the cytotoxic effects of methotrexate (MTX) and doxorubicin (DOX) on PLC/PRF/5 cells. Moreover, the expression of 11 important genes involved in MDR was performed by semi-quantitative reverse transcriptase-polymerase chain reaction (RT-PCR).

**Results:**

PLC/PRF/5 cells (Alexander) were sensitive to DOX and normally resist- ant to MTX. In addition, the results obtained from RT-PCR analysis revealed that *β-catenin* expression was significantly reduced and *ABCG2* significantly overex- pressed 4.85 and 3.34 times (P value<0.05) in DOX and MTX treated cells, respec- tively. Furthermore, a considerable expression of *HIF-1α* and *p65* were detected only in MTX-resistant cells.

**Conclusion:**

Anti-cancer drugs may have more than one target in tumor cells. They not only participate in deregulation of Wnt but also alter NF-κB activation. Moreover, *HIF-1α* was the only anti-apoptotic protein that was significantly induced in the chem- oresistant cells.

## Introduction

Hepatocellular carcinoma (HCC) is the most prevalent adult liver malignancy, the fifth most common cancer and the third cause of cancer deaths worldwide (1). Anthracyclines with a complicated mechanism in HCC therapy are among the most effective and well-known anticancer drugs. Some of the primary derivatives of this class of drugs, including doxorubicin (DOX) and daunorubicin (DNR), were isolated from *Streptomyces peucetius* (2). Methotrexate (MTX), a chemotherapeutic agent, is another common drug in treatment of various cancers including non-Hodgkin’s lymphoma, osteosarcoma, breast and liver cancers (3,4). This compound, like other 4-aminofolate analogues, exerts its cytotoxic effects through competitive inhibition of hydrofolate reductase and thus leads to an intensive drop in intracellular levels of tetrahydrofolate and tymidilate, disrupting the de novo pathway of purine synthesis and consequently inhibiting DNA replication (5,6). However, chemoresistance associated with HCC is the main cause of poor management of this malignancy (2). 

Based on the preliminary results obtained, it was shown that PLC/PRF/5 cells are naturally sensitive to DOX and resistant to MTX, and according to several studies, alteration in MTX uptake, MTX poly-glutamilation, hydrolysis of MTX polyglutamates and their interaction with di-hydrofolate reductase are considered as the common reasons in MTX resistance (3,5). Moreover, it has been demonstrated that administration of some of the estrogenic hormones and genomic instability have potential genotoxic and carcinogenic effects and could specially lead to multi-drug resistance (MDR) (3,4). Therefore, analysis of the main molecular parameters of MTX resistance may lead to better clinical assessment and also prove useful for treatment of the progressive liver cancer. 

Cancer stem cells (CSCs) were recently introduced as a new target in advanced tumor therapy (7). Indeed, stem cells are commonly found in different tissues, even in tumors and have the self-renewal and dye-exclusion abilities which result from the hyper-activated Wnt pathway and subsequently high expression of specific (ATP-binding cassette) ABC transporters such as multi-drug resistance1 (*MDR1*) and ATP-binding cassette sub-family G member 2 (*ABCG2*), both of which are involved in MDR (7). Furthermore, chemotherapy resistance, hypoxia and apoptosis stimuli may lead to the identification and screening of chemical substances (8,9) that target the main signaling pathways such as anti-apoptotic kinase (AKT) and nuclear factor kappa-light-chain-enhancer of activated B cells (NF-κB) in CSCs, and thus could be exploited in mono and combination therapy (10,11). 

The regular canonical Wnt signaling pathway was reported to play an essential role in selfrenewal ability in skin and the gastrointestinal tract with *LGR5*, a specific marker in distinguishing all types of epithelial cells, being one of the target genes of the Wnt/B-catenin pathway (11). It has also been stated that inhibition of this pathway could be a useful strategy to overcome drug resistance (12,14). Moreover, it was observed that the aberrant activation of NF-κB and subsequent altered expression of anti-apoptotic genes associated with this pathway may be responsible for MDR (8,9). 

Since identification of the main factors involved in drug resistance (e.g. deregulation of NF-κB and Wnt signaling pathways) has the potential to screen individuals sensitive to liver cancer therapy, the main purpose of this study was to investigate the impact of traditional chemotherapy on the mentioned pathways by assessing their major molecular parameters. 

## Materials and Methods

### Drug preparation and cell culture

In this experimental study, 50 mg/ml MTX and 2 mg/ml DOX were purchased from Helale ahmar pharmacy (Iran). MTX was also purchased from Sigma (Sigma, USA) and dissolved in 0.5% sodium bicarbonate (Merk, Germany). 

PLC/PRF/5, a hepatocellular cell line, was purchased with NIH-3T3 cell line and human primary neuroblastoma cells from Pasteur Institute of Iran. The cells were cultured in RPMI 1640 supplemented with 10% fetal bovine serum (FBS, Gibco, USA), 35 µg U/ml penicillin (Sigma, USA), 50 µg/ml streptomycin (Sigma, USA) and 2 mg/ml sodium bicarbonate (Merk, Germany), and kept in a humidified atmosphere with 5% CO_2_ at 37˚C. The cell-harvesting process was performed by 0.25% trypsin solution (Sigma, USA) with 0.03% Ethylene di-amine tetra-acetic acid (EDTA, Sigma, USA). 

### Cytotoxicity determination of methotrexate and doxorubicin by methylthiazol tetrazolium

(MTT) assay About 5000 PLC/PRF/5 cells were cultured in 96-well plates. After 24 hours, cultured cells were separately treated with different concentrations of the above mentioned drugs and incubated at 37˚C. Some wells were left untreated and served as controls. After the stipulated time of drug exposure, the culture medium was discarded and cells were incubated with 5 mg/ml MTT solution for 4 hours. The staining solution was then removed and the intracellular MTT was dissolved in 25 μl glycine sorenson buffer with 150 μl dimethyl sulfoxide (DMSO, Sigma, USA). The absorbance of the wells was read at 570 nm by Elisa reader GEN5 (Bio TEK). 

### Apoptosis detection by simultaneous acridine orange/ethidium bromide (AO/EtBr) and Hoechst staining 

About 10000 cells were seeded in 35 mm Petri dishes (Nunc™ Dishes) and incubated under culture conditions for 24 hours. After treatment with different concentrations of the drugs, the plates were incubated 48 hours and 72 hours for DOX and MTX treatment, respectively. 

For cell fixation, 4% paraformaldehyde (PFA)/4% sucrose in phosphate buffer (PBS) was added to the Petri dishes for 15-45 minutes. The cells were then washed with 0.1% triton X-100 (Sigma,USA) in PBS for 5 minutes. First of all, we stained the fixed cells with 2.5 µg/µl AO/EtBr (Sigma, USA) in PBS/0.1% triton X-100 for 5 minutes. Afterwards, the cells were stained with 2.5 µg/µl Hoechst 33342 (Sigma, USA) in PBS/0.1% triton X-100 and checked by fluorescence microscopy (Axoscope 2 plus fluorescence microscopy-ZEISS, software Infinity capture, Germany) (15). 

### Determination of chemosensitivity by diphenylamine and DNA fragmentation assays

The cell culture and treatment steps were performed exactly identical to AO/EtBr and Hoechst staining. After completion of the incubation period, without removing the media, the cells were detached by scraping and transferred to a Falcon tube for cold centrifugation at 5000 rpm. Diphenylamine assay was performed according to the Gercel-Taylor method and the absorbance of the samples was read at 600 nm by Elisa reader GEN5 (Bio TEK, USA) (16). 

For detection of DNA fragments, we repeated the cell culture and treatment steps. After harvesting the cells with their medium, DNA was extracted using a modified extraction buffer, isopropanol and ethanol (Sigma, USA) (17). The appropriate amount of the extracted DNA with ladder was then separately loaded on a 1.5% agarose gel and run at 110 V for 30 minutes. Finally, DNA fragments were detected by EtBr under ultraviolet transilluminator equipped with camera (18). 

### Evaluation of chemoresistance and chemosensitivity by the colony-formation assay

A sufficient number of PLC/PRF/5 cells were cultured and treated similar to AO/EtBr and Hoechst staining. We left aside the treated cells without changing the medium for 2 weeks. The supernatant was slowly removed from the plates and the colonies were then stained with 5 ml 0.01% crystal violet (CV) for 45 minutes. The CV solution was subsequently removed and the plates were eluted with PBS. Finally, the colony counting was performed by ImageJ according to the Tom C. Karagiannis method (19). 

### Evaluation of gene expression by semi-quantitative reverse transcriptase-polymerase chain reaction

After cell culture and treatment (in 35 mm Petri dishes), we extracted RNA by a modified extraction buffer, isopropanol and ethanol (17), and synthesized cDNA through Transcriptor First Strand cDNA Synthesis Kit (Roche, Switzwerland). Polymerization chain reaction (PCR) was carried out with 2x PCR Master Mix (Promega, USA), with adding the required amount of cDNA, 0.5 μl of the primers (10 pM) and ddH2O to a total volume of 20 μl. Initial denaturation and final extension were 95˚C, 4 minutes and 72˚C, 5 minutes, respectively. Three steps of the PCR cycles were denaturation for 30 seconds at 95˚C, annealing step (Table 1) and extension for 60 seconds at 72˚C. The PCR products with a DNA ladder (Fermentas, USA) were separately loaded on a 2% agarose gel and the bands were visualized by EtBr under UV transilluminator equipped with camera. Finally, the intensity of each band was determined by ImageJ software and normalized with β-2-microglobulin (β2M) as the house-keeping reference gene (12).

### Statistical analysis

The data analysis was performed by SPSS version 17.0 ( (IBM Corporation, USA) with significant P<0.05. The results were presented as mean ± SEM and statistical significance was assessed by ANOVA with Tukey’s method (HSD). 

**Table 1:** Details of RT-PCR for all genes studied


Product name	Primer sequence	ta*/time(s)	Cyclenumbers	Amplicon size(bp)

*β-catenin*	F:5´-GAAACGGCTTTCAGTTGAGC-3´	62.1/60	40	166
	R:5´-CTGGCCATATCCACCAGAGT-3´			
*LGR5*	F:5´-TGCTGGCTGGTGTGGATGCG-3´	62.1/60	40	241
	R:5´-GCCAGCAGGGCACAGAGCAA-3´			
*ABCG2*	F:5´-CACAAGGAAACACCAATGGCT-3´	54/45	40	70
	R:5´-ACAGCTCCTTCAGTAAATGCCTTC-3´			
*MDR1*	F:5´-TGATGACCCTAAAAACACCACTG-3´	56/30	40	81
	R:5´-GAACCTATAGCCCCTTTAACTTGA-3´			
*OCT-4*	F:5´-GGGAGATTGATAACTGGTGTGTT-3´	54/45	40	144
	R:5´-GTGTATATCCCAGGGTGATCCTC-3´			
*BCL2*	F:5´-ACAACATCACAGAGGAAGTAGAC-3´	53/45	40	173
	R:5´-ATTCTTGGACGAGGGGGTGT-3´			
*BIRC7*	F:5´-GGGACCCGTGGGAAGAAC-3´	52/45	40	491
	R:5´-CACGCCAAGCAAGGGCCT-3´			
*p50*	F:5´-CACCTAGCTGCCAAAGAAGG-3´	55/45	40	309
	R:5´-AGGCTCAAAGTTCTCCACCA-3´			
*p65*	F:5´-GGCCATGGACGAACTGTTCCC-3´	52/45	40	249
	R:5´-GGAGGGTCCTTGGTGACCAG-3´			
cREL**	F:5´-GCAGAGGGGAATGCGTTTTAG-3´	52/45	40	97
	R:5´-AGAAGGGTATGTTCGGTTGTTG-3´			
*HIF-1α*	F:5´-GAACGTCGAAAAGAAAAGTCTCG-3´	53/60	40	124
	R:5´-CCTTATCAAGATGCGAACTCACA-3´			
*β2M*	F:5´-CGCTCCGTGGCCTTAGC-3´	59/45	40	67
	R:5´-GAGTACGCTGGATAGCCTCCA-3´			


*; Annealing temperature and **; Homo sapiens v-rel reticuloendotheliosis viral oncogene homolog (avian) (REL).

## Results

### There was no significant cytotoxic effect of methotrexate on PLC/PRF/5 cells

**Fig.1:**
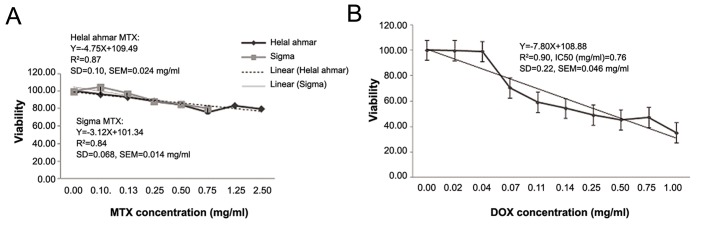
The results of MTT assay. A. No significant cytotoxic effect of MTX (sigma and Helal ahmar) was observed on PLC/PRF/5 cells and B. Significant cytotoxic effect of DOX on PLC/PRF/5 cells especially in high concentrations (>700 µg/ml). MTX; Methotrexate and DOX; Doxorubicin.

MTX efficiency was examined on the NIH- 3T3 cell line and human primary neuroblastoma cells. The results showed that MTX was cytotoxic for these cells and caused damage even in very low concentrations (data not shown). MTT assay showed that PLC/PRF/5 cells were resistant to MTX and sensitive to DOX as the SEMs were 0.014 mg/ml, 0.023 mg/ml and 0.046 mg/ml for Helal ahmar MTX, Sigma MTX and DOX treated cells, respectively. The data analysis showed that there was a significant correlation between untreated and MTX-treated cells while DOX-treated cells had a considerable difference with their control. In addition, the value of the half maximal inhibitory concentration (IC50) of DOX was 0.76 ± 0.046 mg/ml (Fig .1).

### No apoptotic cells was detected in methotrexate-treated PLC/PRF/5 cells by using simultaneous staining of Hoechst 33324 and AO/EtBr 

**Fig.2:**
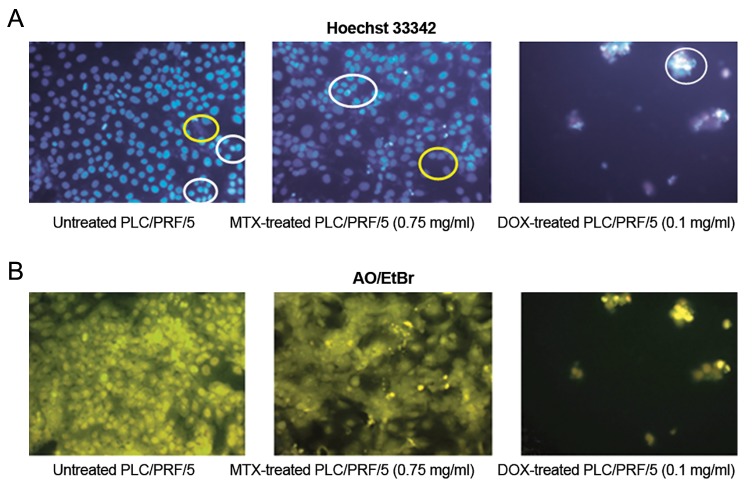
Simultaneous AO/EtBr and Hoechst staining of cells. A. Hoechst staining: the white circles represent the compact DNA with light phase belonging to either the cells with dye-exclusion ability (drug resistant cells) or apoptotic bodies (DOX-sensitive cells). Yellow circles show the relaxed dark blue nuclei of the normal cells in interaction with Hoechst 33342. These cells are differentiable in untreated and MTX-treated cells and B. AO/EtBr staining: it discriminated drug resistant from apoptotic cells with regard to Hoechst staining. The apoptotic cells are orange or red but the drug resistant cells similar to normal cells are green. AO/EtBr; Acridine orange/ethidium bromide, MTX; Methotrexate and DOX; Doxorubicin.

We showed that MTX-treated cells and untreated cells were similar in the shape and number by simultaneous staining of Hoechst 33324 and AO/ EtBr while DOX-treated cells were significantly different from their controls, mostly being apoptotic (Fig .2). 

### Assessment of chemosensitivity by diphenylamine and DNA fragmentation assay 

**Fig.3:**
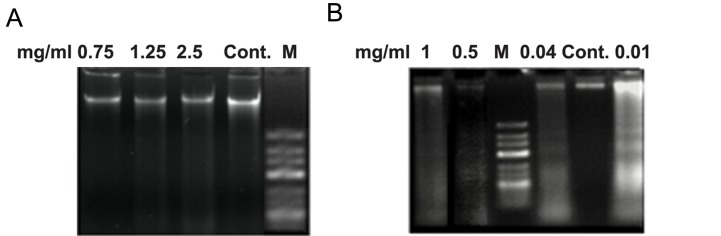
Analysis of DNA Fragmentation on 1.5% agarose gel. A. A unique band was detected in PLC/PRF/5 cells treated with different concentrations of MTX similar to untreated cells and B. The detected DNA fragments in the treated PLC/PRF/5 by different concentrations of DOX. M; Marker, Cont; Control or untreated cell, MTX; Methotrexate and DOX; Doxorubicin.

We determined the percentage of DNA fragmentation 98.50, 97.78, 84.08, 61.50 and 39.20% for 1, 0.5, 0.1, 0.04 and 0.01 mg/ml of DOX, respectively, and 8.37, 7.90 and 6.82% for 2.5, 1.25 and 0.75 mg/ml of MTX respectively. Unlike for MTX, DOX-treated cells showed a considerable difference with untreated cells. These values were in agreement with the detection of DNA fragments on 1.5% agarose gel (Fig .3). 

### Determination of colony-forming ability in methotrexateand doxorubicin-treated PLC/

**Fig.4:**
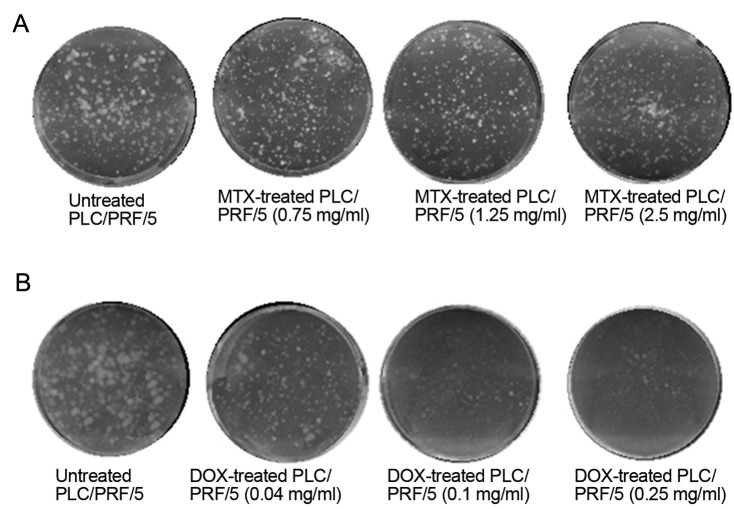
The results of colony-forming assay. Analysis of colony counting by one-way ANOVA test indicated that A. There was no significant difference between untreated and MTX-treated cells and B. There was a significant difference between untreated and DOX-treated cells in colony-forming ability. MTX; Methotrexate and DOX; Doxorubicin.

PRF/5 cells The SEM values of colony counting were 4.62 and 11.59 for control and MTX treatment , respectively. No significant difference was found between colony-forming ability of MTX-treated and control cells (P=0.067). However, the SEMs of colony counting were significantly different between DOX-treated and control cells (33.29 and 8.54 respectively, P=0.001, Fig .4). 

### Evaluation of the well-known Wnt signaling elements in methotrexate-resistant and doxorubicin-sensitive cells

**Fig.5:**
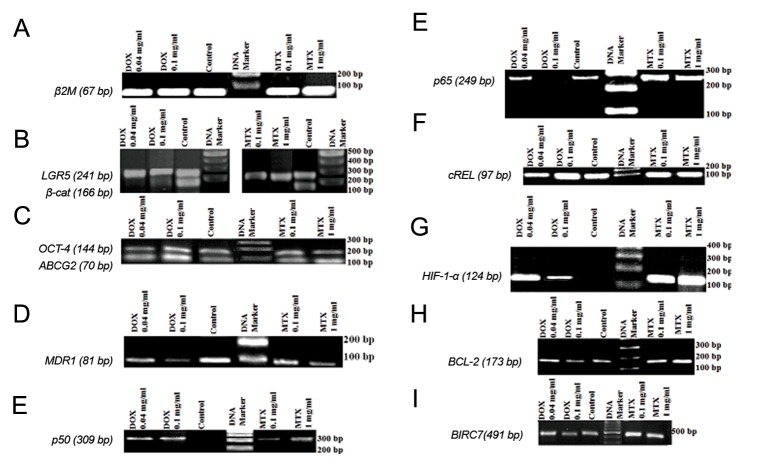
Detection of the expression of Wnt and NF-κB pathway core members in MTX and DOX treated cells on 2% agarose gel. A. β2M was analyzed as the reference gene. The expression of *β-catenin*, as a central protein in the canonical Wnt pathway with its introduced target genes, B. *LGR5*, C. *OCT-4*, D. *ABCG2* and *MDR1*, E. the expression of *p50*, F. *p65*, G. *cREL* as the main transcription factors in NF-κB signaling pathway with their target genes, H. *HIF-1α*, I. *BCL-2* and J. *BIRC7*. MTX; Methotrexate and DOX; Doxorubicin.

**Fig.6:**
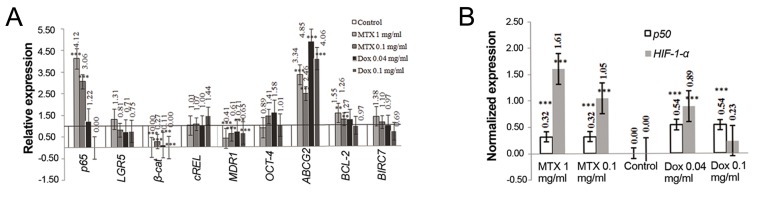
The expression alterations of Wnt and NF-κB pathway core members in MTX-resistant and DOX-sensitive cells. A. The expression alteration of genes which had expression in the control cells and B. The alteration of expression of *p50* and *HIF-1α* which did not have any expression in the control cells. *; P<0.05, **; P<0.01, ***; P<0.001, MTX; Methotrexate and DOX; Doxorubicin.

According to our findings (Fig .5), *β-catenin* expression was significantly reduced by 86.24 and 94.5% in MTXand DOX-treated cells respectively. However, there was no significant difference in *LGR5* expression between the untreated and the treated cells. Furthermore, we detected a considerable downregulation in *MDR1* expression by 49.06 and 32.07% in MTXand DOX-treated cells respectively. We also observed that *ABCG2* expression was significantly upregulated 1.94-fold in MTX-treated and 3.5-fold in DOX-treated cells (Fig .6A). Finally, we did not detect any significant difference for *OCT-4* expression between either of the treated cells with their controls. 

### Evaluation of the well-known NF-κB elements in methotrexate-resistant and doxorubicin-sensitive cells

Although we observed no significant difference in *cREL* expression preand post-treatments, MTX and DOX could significantly induce *p50* expression. In addition, *p65* expression was considerably upregulated (2.58-fold) in MTX-treated cells; however, its expression was reduced by 38.5% in DOX-treated cells (Fig .5). 

*HIF-1α* was expressed in the presence of the treatments specially in MTX-resistant cells but DOX could significantly induce *HIF-1α* expression similar to MTX only in low concentration (400 µg/ml). Although, *BCL-2* expression was upregulated in MTX-treated cells with a significant difference at the 0.01 level, its expression was reduced to 17% in DOX-treated cells. Moreover, a considerable expression of *BIRC7* was detected in MTX-resistant and DOX-sensitive cells similar to *BCL-2* (Fig .6A) but its alteration was not significant at the 0.05 level (Fig .5). 

## Discussion

MTX and 5-floruracil (5-FU), both antifolate compounds, (20) and DOX, an anthracycline, are commonly used in cancer therapy, however, their efficacy is limited due to MDR (2). Considering the weak response of these drugs in advanced tumors even in combination with other anti-cancer drugs, it seems to be essential for cancer management to identify important molecular elements in tumor resistance against these drugs (20). 

Various factors such as drug inactivation or exclusion and alteration of the molecular targets of chemotherapeutic agents contribute to chemotherapeutic resistance (21). They are probably involved in altered activation of several molecular factors including the putative CSC markers (*LGR5*, *NANOG* and *OCT-4*), Wnt and NF-κB signaling pathways as well as the expression of anti-apoptotic proteins (5,20). Therefore, in the present study we evaluated some of the parameters that are assumed to affect chemotherapeutic resistance in PLC/PRF/5 cells. 

Current studies have targeted CSCs, particularly in HCC management (22). In this study, PLC/ PRF/5 was selected as an aggressive liver cancer cell line due to hyper-activation of Wnt pathway and subsequent expression of CD133 as a wellknown CSC surface marker (10,13,23). Furthermore, these tumor cells are involved in overexpression of certain oncogenic proteins including *c-MYC* and *c-RAS* and also carry a homozygous point mutation in hotspot codon 249 (GC→TA) of p53 (24). The results of MTT assay, AO/EtBr and Hoechst staining, DNA fragmentation and colony formation indicated that these cells are naturally resistant to MTX and sensitive to DOX. 

Based on our findings, Wnt signaling pathway was downregulated in MTX-resistant and DOXsensitive PLC/PRF/5 due to a significant decrease in *β-catenin* expression along with a reduction of *LGR5* expression in DOX-treated cells only. Furthermore, with respect to the considerably high expression of *LGR5* and *OCT-4* in MTX-treated cells, it seems that MTX acts as an inducer of epithelial to mesenchymal transition (EMT) phenotype in PLC/PRF/5. Besides, *ABCG2* was observed to be significantly overexpressed after both treatments, which is consistent with the results of several similar studies (25,29), however, we found a considerable downregulation of *MDR1* in treated cells. These results demonstrated that although conventional chemotherapeutic agents were used to act as inhibitors of Wnt pathway, they are impotent against MDR. 

Activation of NF-kB pathway is under strict control in normal cells. However, there are some molecular elements that induce aberrant activation of this pathway (30) and subsequently lead to a wide range of inflammatory and auto-immune diseases as well as cancers (31). 

Some of the former earlier reports have revealed that either *p50* overexpression or its enhanced DNA binding affinity lead to reduced expression of NF-κB-dependent genes, while *p65* plays a key role in oncogenic activity of NF-kB and subsequent expression of anti-apoptotic proteins, including *survivin, IAPs, Cyclin D1, c-MYC, BCL-2* and *HIF-1α* (30,32,34). In accordance with these studies, we detected *p50* expression but no significant alteration in *cREL* expression in both DOXsensitive and MTX-resistant cells. In addition to a considerable level of *p65* expression, *BCL-2* and *BIRC7* genes were also expressed in both treated and untreated cells. Furthermore, we detected HIF1α expression merely in the treated cells, though it was reduced upon *p65* downregulation caused in turn by an increase in DOX dose. 

Considering i. The significant expression of *BIRC7* and *BCL-2* in DOX-treated cells and similarly in MTX-treated cells, ii. The result of MTT assay and iii. Findings mentioned above, it is assumed that cells act in a dose-dependent manner in response to DOX treatment where partial resistance is observed in low concentrations of this drug and sensitivity at a high dosage (accompanied with *p65* downregulation). 

These findings suggest that *p65* expression may be a necessary condition for expression of antiapoptotic proteins such as BRIC7 and *BCL-2* in the drug resistant cells; however the presence of the drug was a sufficient condition for having a remarkable expression of *HIF-1α* as a probable prognostic parameter for MDR, especially in low doses of the chemotherapeutic agents. Altogether, simultaneous alterations observed in the activity of Wnt and NF-κB pathways in the presence of these treatments suggest that there may be either a crosslink between these two critical signaling pathways or that a drug could have multiple molecular targets in the cells. 

## Conclusion

Further qualitative and quantitative assays for protein evaluation in the affected tissues of drug resistant HCC patients along with *in vivo* experiments are required to confirm these data. However, determination of common factors involved in resistance to chemotherapy or radiotherapy such as *HIF-1α* could help oncologists to select an appropriate (combinatorial) therapy for cases with advanced cancers. 
